# *Beta-hemolytic Streptococci* in respiratory illness

**DOI:** 10.6026/9732063002001495

**Published:** 2024-11-05

**Authors:** Gayathri Gunasekaran, Dinesh Kumar Thirugnanam, N. Nalini Jayanthi

**Affiliations:** 1 Department of Microbiology, SRM Medical College Hospital and Research Centre, Kattankulathur-603 203, Tamil Nadu, India; 2 Department of Microbiology, Meenakshi Ammal Dental College and Hospital, Meenakshi Academy of Higher Education and Research (Deemed to be university), Chennai, Tamil Nadu, India; 3 Department of Respiratory Medicine, SRM Medical College Hospital and Research Centre, Kattankulathur-603 203, Tamil Nadu, India

**Keywords:** *Beta-hemolytic Streptococci*, respiratory illness

## Abstract

*Beta-hemolytic Streptococci* are associated with various respiratory illnesses, such as pharyngitis, scarlet fever and pneumonia, highlighting the need for enhanced diagnostic, therapeutic and preventive strategies. Advances in immunology and molecular biology have provided insights into the etiology and host immune responses to these infections, but many questions remain unanswered. Further research is needed to develop advanced diagnostics, explore vaccine candidates, understand immune responses and address antibiotic resistance. Epidemiological studies are crucial to improving our understanding of these infections and their public health impact. A multidisciplinary approach integrating epidemiology, microbiology, immunology and clinical medicine is essential to reduce the burden of beta-hemolytic streptococcal infections and improve overall treatment and prevention efforts.

## Background:

*Beta-haemolytic streptococci*, a type of bacteria, are primarily found in the human respiratory system and are linked to various respiratory infections, including pharyngitis and tonsillitis. Invasive streptococcal infections, caused by Groups A, C and G, pose significant public health risks due to infection rates, antibiotic resistance and inappropriate antibiotic use. Understanding the characteristics and antibiotic susceptibility profiles of these germs is crucial for effective clinical care [1]. In India, antimicrobial resistance is a challenge in treating infections, especially Group A (GAS), which is linked to pharyngitis and skin infections. Oral streptococci exhibit variable complement immune resistance and poorly understood systemic infection mechanisms. Understanding the frequency and determinants of these resistance patterns is crucial for effective management [2]. The complex dynamics of host-pathogen interactions in the respiratory system highlight the immune system's role in both resistance and vulnerability to infections caused by beta-haemolytic streptococci. Some strains of GAS induce inflammation, leading to post-streptococcal glomerulonephritis (PSGN), which can cause rheumatic fever, rheumatic heart disease and PSGN following skin or throat infections [3]. Developing a safe and effective vaccine for GAS is crucial, but it must overcome challenges like antigen variability and regional strain differences. Effective immunization regimens must include age groups and efficacy requirements to reduce antibiotic usage [4]. The intricate relationships that arise between respiratory infections, beta-haemolytic streptococci and the immune system of the host are examined in this publication. By carefully reviewing the relevant literature, we will provide light on the molecular and cellular mechanisms behind the development and resolution of respiratory infections brought on by beta-haemolytic streptococcal bacteria.

## Classification and characteristics:

*Beta-hemolytic Streptococci* are a class of bacteria known for their role in respiratory illnesses and human immunity [5]. They are primarily classified by their Lancefield group antigen, which identifies the specific polysaccharide present in the bacterial cell wall. *Streptococcus pyogenes*, a significant member of the BHS, is known for its ability to cause a wide range of clinical signs, from minor pharyngitis to major invasive infections [6]. BHS are catalase-negative and produce two main hemolysins, streptolysin O and streptolysin S, contributing to their hemolytic activity and cytotoxic effects [7]. They are common causes of acute pharyngitis, scarlet fever, necrotizing fasciitis, streptococcal toxic shock syndrome and bacteraemia. Understanding the morphological and biochemical characteristics of *Beta-hemolytic Streptococci* is crucial for accurate diagnosis and effective management of respiratory infections and associated immune responses.

## Respiratory infections caused by beta hemolytic streptococci:

*Beta-hemolytic streptococcus* pyogenes (BHS) is a group of bacteria that cause a range of respiratory ailments, including tonsillitis, sinusitis, pneumonia and pharyngitis. Pharyngitis, a sore throat, is a common BHS symptom, while tonsillitis, inflammation of the tonsils, can lead to complications [8]. Rapid diagnosis is necessary before antibiotics are prescribed. BHS is also linked to acute bacterial sinusitis, characterized by fever, purulent nasal discharge, nasal congestion and facial pain. In severe cases, antibiotics targeting BHS are recommended [9]. BHS is a significant cause of community-acquired pneumonia, with symptoms such as fever, dyspnea, coughing and chest pain [10]. Host immunity plays a significant role in an individual's susceptibility to BHS respiratory infections. Factors such as age, comorbidities, immunocompromised conditions and prior exposure impact the disease's severity and prognosis [11]. Investigating the immune mechanisms behind BHS infections can lead to the development of vaccinations and immunotherapies that target virulence factors or strengthen the host's immune system.

## Pathogenesis:

*Streptococcus pyogenes*, a bacterial disease, causes infections like pharyngitis, skin infections and invasive conditions like necrotizing fasciitis and pneumonia. It attaches to respiratory tissues and penetrates through enzymes and specialized secretion systems [12]. The bacteria can inject toxins and other virulence factors into host cells, compromising cell integrity and promoting invasion. To eliminate the infection, the host initiates an inflammatory response [13]. *Beta-hemolytic Streptococci* (BHS) infections are influenced by virulence factors such as streptolysin, M protein and exotoxins. Streptolysin O and SLS are two forms of the cytotoxin, causing tissue damage and cell lysis [14]. M protein prevents phagocytosis, modifies the host immune response and facilitates adhesion to host cells. Streptococcal pyrogenic exotoxins (SPEs) are exotoxins released by BHS, causing a strong and dysregulated immune response [15]. Overabundance of pro-inflammatory cytokines can lead to tissue death and signs of systemic infection.

## Strain-specific pathogenicity:

*Beta-hemolytic Streptococci*, a group of bacteria with unique virulence traits, are responsible for various respiratory infections [16]. These strains can infiltrate and colonize host tissues, evade immune responses and cause disease. Understanding these virulence factors is crucial for developing precise diagnostic techniques, reducing antibiotic misuse and developing targeted treatment strategies. This knowledge can lead to the development of vaccines and antimicrobial medications, improving patient outcomes and reducing antibiotic resistance.

## Mechanisms of infection:

Bacterial hyphae (BHS) attaches to respiratory epithelial cells due to adhesins, making it easier for bacteria to colonize. Understanding these pathways can help identify potential therapeutic interventions. BHS can evade host immune responses through biofilm formation, immunomodulatory chemicals, or surface protein synthesis [17]. Understanding these strategies is crucial for developing immunotherapeutic approaches against BHS infections. Certain BHS strains produce toxins like superantigens and streptolysins, which contribute to tissue damage and systemic infections [18]. Understanding the molecular interactions between host cells and BHS can provide insights into infection and host defense, including host immunological pathways, genetic variables and BHS cellular processes.

## Clinical manifestations:

BHS respiratory infections, often resembling less serious conditions like tonsillitis or pharyngitis, can cause severe complications like necrotizing fasciitis, acute post-streptococcal glomerulonephritis and acute rheumatic fever [19]. Atypical appearances can complicate diagnosis, as they may not show conventional signs. Asymptomatic carriers pose another challenge, as they do not appear sick but serve as reservoirs for the virus. To prevent BHS spread, asymptomatic carriers must be identified, especially in close-contact environments like schools or hospitals.

## Immune response to beta hemolytic streptococci:

*Beta-hemolytic Streptococci*, particularly Group A Streptococcus (GAS), cause various human illnesses, ranging from simple skin infections to more severe ones like necrotizing fasciitis and streptococcal toxic shock syndrome. The immune response against these bacteria involves innate and adaptive components. The innate immune system, composed of phagocytic cells, breaks down physical barriers, allowing bacteria to enter the body. The complement system, consisting of B cells and T cells, helps neutralize bacteria and increase phagocytes' ability to eliminate them [20]. Memory B and T cells are produced after infection eradication, providing protection against reinfection [21]. However, excessive or dysregulated immune activation can cause tissue damage and contribute to streptococcal illnesses. Certain strains of *Beta-hemolytic Streptococci* can compromise host immune defenses.

## Resistance of the host immune system against *Beta-hemolytic Streptococci*:

*Beta-hemolytic Streptococci* (BHS), particularly Group A streptococcus (GAS), are a significant bacterial pathogen that require both innate and adaptive immunity to effectively eradicate. Innate immune cells, such as dendritic cells and macrophages, express Toll-like receptors (TLRs) to recognize pathogen-associated molecular patterns on BHS surfaces, triggering a series of signaling cascades that boost the immune system and generate pro-inflammatory cytokines [22]. B cells, which produce antibodies in response to BHS antigens, support opsonization, complement activation and bacterial toxin neutralization [23]. T cells, which produce cytokines, regulate inflammation, activate immune cells and promote BHS elimination. A coordinated effort between innate and adaptive immunity is necessary for effective BHS infection elimination and long-term immunological memory formation.

## The challenges and potential long-term implications:

BHS infections, particularly Group A Streptococcus (GAS), can be eradicated by the immune system's reaction, but they can also lead to autoimmune diseases like rheumatic fever [24]. Genetic variables, family history and ethnic origin can influence an individual's vulnerability to autoimmune reactions [25]. Repeated exposure to BHS antigens can result in long-term immune activation and a higher risk of autoimmune responses. Excessive or dysregulated immune activity during BHS infections can lead to tissue damage and inflammation, which are factors in the pathophysiology of autoimmune diseases [26]. Post-streptococcal glomerulonephritis, an autoimmune kidney disease, can also be caused by BHS infections. Environmental factors such as exposure to pathogens, socioeconomic level and healthcare accessibility can also contribute to the development of autoimmune diseases.

## Diagnosis challenges and treatment:

## The current diagnostic approaches:

Diagnostic methods for respiratory infections associated with *Beta-hemolytic Streptococci* (BHS), particularly Group A streptococcus (GAS), often involve clinical assessment, rapid antigen detection tests (RADTs) and confirmatory laboratory testing [27]. Symptoms include a painful throat, fever, tonsillar exudates, enlarged lymph nodes and cough. Rapid Antigen Detection Tests (RADTs) are used for prompt diagnosis, particularly in children [28]. Throat culture is the gold standard for diagnosing streptococcal pharyngitis, but it takes 24 to 48 hours to confirm BHS identity. PCR tests are growing for quick and accurate identification of BHS DNA in clinical specimens [29]. Molecular testing can be helpful when throat culture and RADT results are contradictory. Serological assays can detect post-streptococcal sequelae or determine recent infections. The selection of a diagnosis method depends on clinical presentation, resource availability and local recommendations.

## Antimicrobial strategies and therapeutic interventions for effective management:

To treat Group A streptococcus (GAS) pharyngitis, antimicrobial methods and therapeutic interventions are necessary. Penicillin and amoxicillin are the first-choice medicines, but other antibiotics like macrolides and cephalosporins may be used for those allergic to penicillins [30]. Macrolide resistance among GAS strains is a growing concern and culture and susceptibility testing may be necessary in cases of treatment failure or high macrolide resistance rates [31]. Non-steroidal anti-inflammatory medicines (NSAIDs) like acetaminophen and ibuprofen can help reduce pain, fever and inflammation. Rest and proper water are essential for bolstering the immune system and accelerating healing [32]. Timely diagnosis and management of GAS pharyngitis can reduce the chance of complications, such as post-streptococcal glomerulonephritis and rheumatic fever. Practice respiratory hygiene, stay away from work or school and evaluate and treat close contacts of affected individuals.

## Public health impact:

Group A Streptococcus (GAS) causes significant respiratory infections worldwide, causing pain, missed work and reduced productivity. Major side effects include rheumatic fever and heart valve damage [33]. Drug-resistant infections arise from overuse and misuse of antibiotics. A comprehensive plan for prevention, diagnosis and treatment is needed. Host susceptibility is influenced by age, immune system performance and pre-existing medical conditions.

## The implications for healthcare systems and strategies for prevention:

Strep throat is a common cause of primary care visits, burdening medical resources and increasing treatment costs. Antibiotic-resistant strains can arise from overuse or misuse of antibiotics, making antibiotic stewardship programs crucial [34]. Despite mild symptoms, streptococcal infections can cause severe consequences, including scarlet fever, post-streptococcal glomerulonephritis and rheumatic fever. Public health initiatives like hand washing and staying home can slow transmission [35]. Currently, there is no vaccine for GAS infections, but efforts are being made to develop a vaccine. Effective prevention and control depend on further research on epidemiology, pathophysiology and therapy.

## Antibiotic resistance:

## The rise of antibiotic resistance in beta hemolytic streptococci:

*Beta-hemolytic Streptococci* have become resistant to various antibiotic families due to overuse and misuse in both community and clinical settings. This resistance is facilitated by inappropriate prescribing practices, early termination of antibiotic regimens and the use of antibiotics for animal production [36]. The emergence of antibiotic-resistant *Beta-hemolytic Streptococci* has significant implications for patient treatment and public health, increasing the risk of complications, lengthening illness and increasing healthcare costs. Clinicians face challenges in treating infections caused by antibiotic-resistant streptococci and broad-spectrum antibiotics may be necessary [37]. To address antibiotic resistance, a multimodal approach is needed, including antibiotic stewardship initiatives, public awareness and funding for novel antibiotics and non-traditional treatments.

## Prevention and vaccination:

## Preventive measures, hygiene practices and the role of vaccination:

To prevent *Beta-hemolytic Streptococci* transmission, people should wash their hands with soap and water, especially after touching objects in public spaces or after sneezing or coughing. Covering the mouth and nose with a tissue or elbow can help stop the transmission of respiratory droplets containing streptococcal bacteria [38]. People with streptococcal infections should stay away from close contact and frequently clean and disinfect surfaces. Vaccines targeting Group A Streptococcus (GAS) are being developed, with some showing promise in preclinical and early clinical trials. Public health campaigns and educational programs can increase knowledge of symptoms, treatment value and antibiotic prescription guidelines [39]. To reduce the impact of these illnesses on world health, more research, funding for vaccine development and public health initiatives are needed.

## Exploring novel avenues in vaccine development:

The search for universal Group A Streptococcus (GAS) immunisation remains challenging due to the lack of clinically approved candidates. VAX-A1, a multivalent protein conjugate GAS vaccine, showed potential cross-protection against GBS-related newborn illnesses [40]. However, many GAS vaccine projects focus on targeting the M protein surface epitope. It's important to examine potential secondary effects on infant GBS infection frequency during Phase III clinical trials or post-marketing reviews if these vaccines progress.

## Future directions:

Improved Diagnostic Techniques, Vaccine Development and Understanding Immune Response, Monitor and address antimicrobial resistance, Epidemiological Studies

## Conclusion:

*Beta-hemolytic Streptococci* are linked to respiratory illnesses like pneumonia and chronic lung conditions, necessitating effective diagnostic, therapeutic and preventive strategies. Recent advancements in immunology and molecular biology have improved our understanding of their etiology and host immune responses. Future research should prioritize advanced diagnostics, vaccine development, antibiotic resistance and epidemiological studies to mitigate their impact.
